# Supplementary material for: Motion sickness symptoms during jumping exercise on a short-arm centrifuge
Source: PLoS One. 2020 Jun 11;15(6):e0234361. doi: 10.1371/journal.pone.0234361 (PMC7289365; doi:10.1371/journal.pone.0234361)
Supplement: Attachment — Submitted filename: Response to Reviewer.docx [file pone.0234361.s002.docx]

**Response to Reviewer**

Dear Prof Iwase,

Dear Editor,

Thank you for your helpful comments on our manuscript. We have read them carefully and worked on a revised version that hopefully complies to your requirements. Please find a list of changes based on your input and comments below:

**Editor comments:**

1. The manuscript show repetitive jumping exercises during centrifugation are feasible and tolerated. This would allow future studies to develop training protocols of exercise as countermeasures in artificial gravity. Physiological response of jumping exercises was analyzed, but not included. The authors should follow the journal format referring to an article published. For ex., Introduction should follow Methods (p.8-10) section. Introduction section (p.3) is written verify briefly. They could have provided more details on bone loss and nervous system affected with gravity changes/or under low gravity, ex., “However, bone mass is not maintained”; “…physical exercise may be more efficient.”

**Answers**:

a) Physiological data were assessed by Kramer et al. as they were funded in performing the investigation by the Space Administration of the German Aerospace Center for this. A paper focusing on the jumping performance and the physiological impact have recently been accepted by your journal entitled: “Adaptability of a jump movement pattern to a non-constant force field elicited via centrifugation” (PONE-D-20-00506R1) Authors: Kramer, Kümmel, Dreiner, Willwacher, Frett, Niehoff, Gruber.

b) We changed the article structure and have added more details in the introduction regarding effects of weightlessness on the human body.

2. Please ensure that your manuscript meets PLOS ONE's style requirements, including those for file naming.

**Answer**: We changed the style requirements as requested.

3. We note that you have indicated that data from this study are available upon request. PLOS only allows data to be available upon request if there are legal or ethical restrictions on sharing data publicly.

**Answer**: We will upload an anonymized data set as requested.

4. We note that one or more of the authors are employed by a commercial company: KBRwyle GmbH.

1. Please provide an amended Funding Statement declaring this commercial affiliation, as well as a statement regarding the Role of Funders in your study. If the funding organization did not play a role in the study design, data collection and analysis, decision to publish, or preparation of the manuscript and only provided financial support in the form of authors' salaries and/or research materials, please review your statements relating to the author contributions, and ensure you have specifically and accurately indicated the role(s) that these authors had in your study. You can update author roles in the Author Contributions section of the online submission form.

Please also include the following statement within your amended Funding Statement.

“The funder provided support in the form of salaries for authors [insert relevant initials], but did not have any additional role in the study design, data collection and analysis, decision to publish, or preparation of the manuscript. The specific roles of these authors are articulated in the ‘author contributions’ section.”

If your commercial affiliation did play a role in your study, please state and explain this role within your updated Funding Statement.

2. Please also provide an updated Competing Interests Statement declaring this commercial affiliation along with any other relevant declarations relating to employment, consultancy, patents, products in development, or marketed products, etc.

Within your Competing Interests Statement, please confirm that this commercial affiliation does not alter your adherence to all PLOS ONE policies on sharing data and materials by including the following statement: "This does not alter our adherence to  PLOS ONE policies on sharing data and materials.” (as detailed online in our guide for authors [http://journals.plos.org/plosone/s/competing-interests](about:blank)) . If this adherence statement is not accurate and  there are restrictions on sharing of data and/or materials, please state these. Please note that we cannot proceed with consideration of your article until this information has been declared.

 Please include both an updated Funding Statement and Competing Interests Statement in your cover letter. We will change the online submission form on your behalf.

Please know it is PLOS ONE policy for corresponding authors to declare, on behalf of all authors, all potential competing interests for the purposes of transparency. PLOS defines a competing interest as anything that interferes with, or could reasonably be perceived as interfering with, the full and objective presentation, peer review, editorial decision-making, or publication of research or non-research articles submitted to one of the journals. Competing interests can be financial or non-financial, professional, or personal. Competing interests can arise in relationship to an organization or another person. Please follow this link to our website for more details on competing interests: [http://journals.plos.org/plosone/s/competing-interests](about:blank)

**Answer:** We have double checked authors’ role and have added the following passage to the section regarding funding:

*The study was part of the German National Centrifuge Program 2 financed by the Space Administration of the German Aerospace Center. KBRwyle GmbH provided the salary for D,G but did not have any additional role in the study design, data collection and analysis, decision to publish, or preparation of the manuscript.*

As there are no competing interests and we will share all relevant data and materials we added the following passage to the section regarding competing interests:

*KBRwyle provided the salary for DAG but did not have any additional role in the study design, data collection and analysis, decision to publish, or preparation of the manuscript.*

**Reviewer comments:**

1. The scheme of jumping during acceleration is very difficult to read from the text. They should show the photos or drawings of the jumping performance.

   Head movements during centrifuge are very difficult to understand from the text. The authors should show the photos or drawings, or the recordings of accelerometer of the head of the subject should be shown in relation with the three-dimensional gravitational acceleration. Fig. 3 is a transition in the time course, but the actual drawing of three-dimensional G transition is requested.

**Answer:** We understand the difficulties to understand the movements. We have added explanation about the sledge functionality in figure 1. Furthermore we included an additional figure (figure 2) that shows a drawing of a participant on the centrifuge in bird´s-eye perspective.

2. The unit of gravitational acceleration should be the capital G italic to distinguish it from the gram unit of mass, and a space is necessary between numeral and G.

   The authors should use the +Gz and −Gz in reference to the body axis i.e. +Gz as the gravity of head to the foot, and −Gz as the foot to the head, of the subject, not in terrestrial +/−Gz.

   Comma and period are mixed in the method of displaying the decimal point. It should be unified to either, and preferably to a period in the English sentence.

**Answer:** We have double-checked and corrected the punctuation in accordance with your comments and have added a more precise description of the centrifuge protocols. Furthermore we change the description of the different protocols to avoid further misinterpretation.

3. Motion sickness scores and angular accelerations of head movements should be expressed by graphs not tables.

**Answer**: We plotted the MSAQ scores and the head movement velocities, see figures 4 and 6.

4. Please show the rationale to separate the sessions at least three days.

**Answer**: We added the passus: “…to allow for muscle recovery” to the manuscript. Jump exercises in supine position in an alternating gravity level were novel to the subjects therefore we planned at least three days for recovery.

5. As far as the reviewer know, the name of the facility is Envihab, not :envihab, did they change the name?

**Answer**: We used the naming in accordance with the description on the DLR homepage (please see: <https://www.dlr.de/envihab/en/desktopdefault.aspx>).
